# Supplementary material for: The interactome of intact mitochondria by cross-linking mass spectrometry provides evidence for coexisting respiratory supercomplexes
Source: Mol Cell Proteomics. 2017 Dec 8;17(2):216–32. doi: 10.1074/mcp.RA117.000470 (PMC5795388; doi:10.1074/mcp.RA117.000470)
Supplement: Supplemental Data [file supp_RA117.000470_133922_0_supp_23978_fzffwf.zip › spectra_annotation/mito_DR_spectra_annotation/19-1-4-1-129-1.pdf]

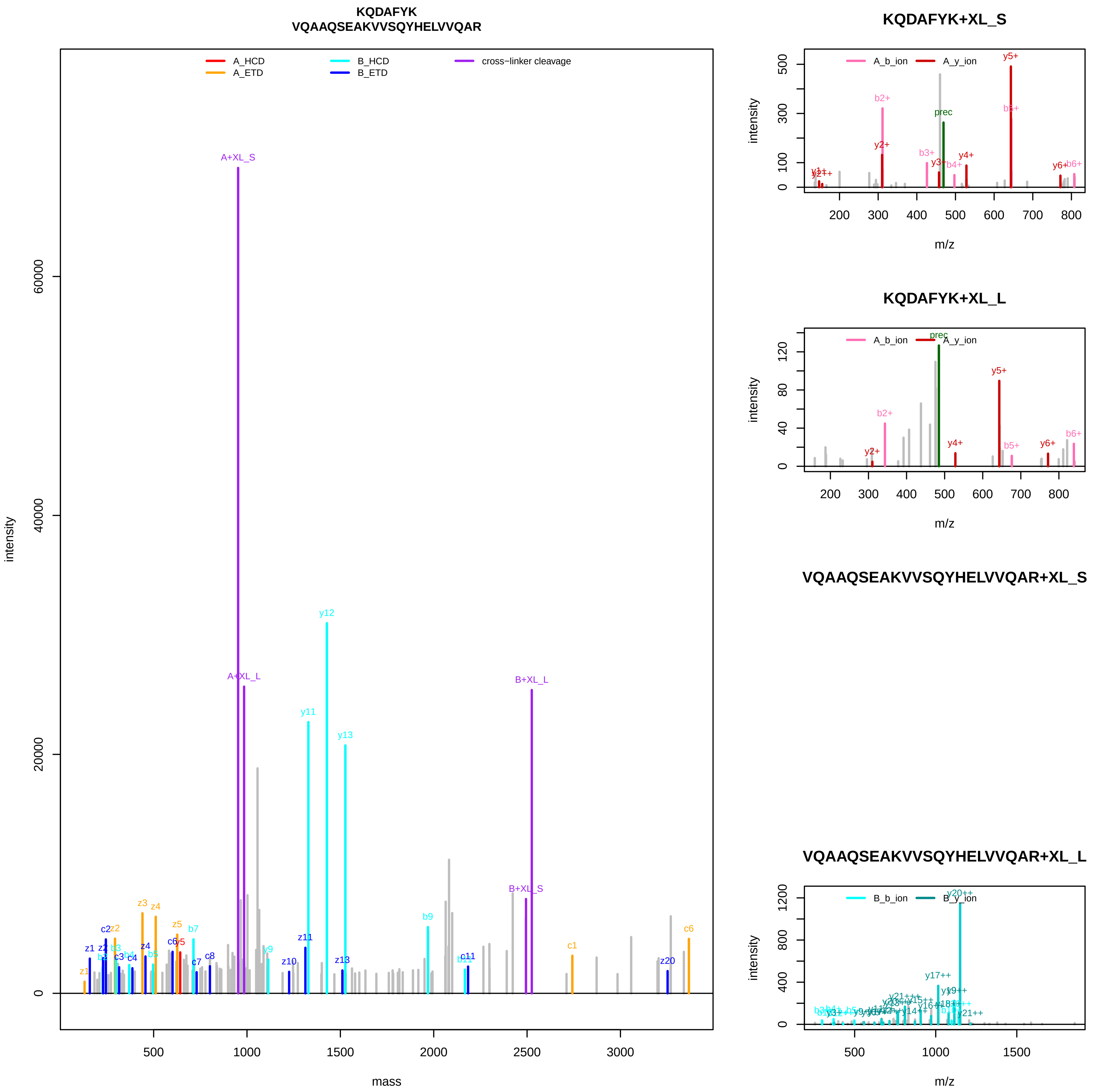

KQDAFYK
VQAAQSEAKVVSQYHELVVQAR
A_HCD
A_ETD
B_HCD
B_ETD
cross−linker cleavage
A+XL_S
A+XL_L
B+XL_L
B+XL_S
y12
y11
y13
b9
z1
z2
c2
b2
b3
c3
c4
b4
z3
z4
b5
c6
y5
z5
b7
c7
c8
y9
z10
z11
z13
b11
c11
c1
z20
c6
intensity
mass
0
20000
40000
60000
500
1000
1500
2000
2500
3000
KQDAFYK+XL_S
A_b_ion
A_y_ion
y5+
b2+
prec
b5+
y2+
b3+
y3+
b4+
y4+
y6+
b6+
intensity
m/z
KQDAFYK+XL_L
VQAAQSEAKVVSQYHELVVQAR+XL_S
VQAAQSEAKVVSQYHELVVQAR+XL_L
B_b_ion
B_y_ion
y20++
y17++
y19++
y21++
